# A Low-Cost Digital Microscope with Real-Time Fluorescent Imaging Capability

**DOI:** 10.1371/journal.pone.0167863

**Published:** 2016-12-15

**Authors:** Md. Mehedi Hasan, Mohammad Wajih Alam, Khan A. Wahid, Sayem Miah, Kiven Erique Lukong

**Affiliations:** 1 Department of Electrical and Computer Engineering, University of Saskatchewan, Saskatoon, Canada; 2 Department of Biochemistry, College of Medicine, University of Saskatchewan, Saskatoon, Canada; Consiglio Nazionale delle Ricerche, ITALY

## Abstract

This paper describes the development of a prototype of a low-cost digital fluorescent microscope built from commercial off-the-shelf (COTS) components. The prototype was tested to detect malignant tumor cells taken from a living organism in a preclinical setting. This experiment was accomplished by using Alexa Fluor 488 conjugate dye attached to the cancer cells. Our prototype utilizes a torch along with an excitation filter as a light source for fluorophore excitation, a dichroic mirror to reflect the excitation and pass the emitted green light from the sample under test and a barrier filter to permit only appropriate wavelength. The system is designed out of a microscope using its optical zooming property and an assembly of exciter filter, dichroic mirror and transmitter filter. The microscope is connected to a computer or laptop through universal serial bus (USB) that allows real-time transmission of captured florescence images; this also offers real-time control of the microscope. The designed system has comparable features of high-end commercial fluorescent microscopes while reducing cost, power, weight and size.

## Introduction

Fluorescence microscopy is a powerful and popular technique used by modern biologists, especially neurobiologists as it allows them to distinguish between the object of interest to those of not interest. Because of its inherent selectivity, it has become a very important part of microscopy in the field of biology. Due to the large spectral range of fluorophores, it is often possible to image different cellular, subcellular and molecular structures simultaneously [1]. Also, the intrinsic fluorescent products (such as, green fluorescent protein and its bi-products) allows biologists to genetically tag protein of living beings which leads us into a new era of fluorescence microscopy. Hence, the rapidly advancing innovation of microscopes leads to the development of innovative fluorescence techniques, due to which, it is now possible to see microscopic structures even in deep tissues in three dimensions [2]. Fluorescence microscopy is commonly used in modern laboratories to view, localize, and track single fluorescing particles in a wide selection of organic and inorganic structures. This is why fluorescence imaging has found its place in a variety of applications including water quality estimation [3, 4], food quality estimation [5], molecular imaging [6], optical biopsy [7] in gastrointestinal endoscopy, and many others.

However, for a person who is inexperienced in fluorescent microscopy, matching the best approach to the biological experiment can be difficult. Optimal and efficient use of fluorescence microscopy demands a basic knowledge of strengths and weaknesses of the different approaches as well as an understanding of the elementary trade-offs of the variables related to it. Familiarity with the principle of fluorescence is a prerequisite for taking advantage of many of these technological advancements towards fluorescence microscopy. One way forward is to start exposing the students (or learners) at schools and colleges to different fluorescent approaches. However, the size, cost and complexity of current fluorescence microscope requires sophisticated laboratories that are well funded and have trained personnel to maintain and operate it [8, 9]. Hence, it is still a challenge to equip modern schools and colleges with these expensive microscopes which makes it difficult to get hands on experience with this equipment for many students (or learners) at these levels.

As a result, even if the students have sound theoretical knowledge, they lack in practical knowledge. Therefore, the objective is to design a low-cost fluorescence microscope to be used in schools and academic institutions. In this research, we have designed a prototype of a fluorescence microscope using inexpensive commercial off-the-shelf components that is digital in nature and has real-time diagnostic capability.

## Materials and Methods

**Fig 1:**
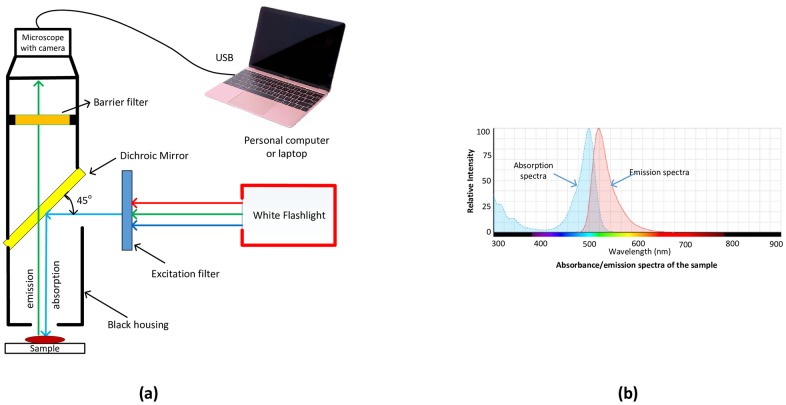
(a) Block diagram of the fluorescence microscope; (b) absorbance/emission spectra of the sample under test (adapted from [10]). The excitation filter passes through lights of 400-499nm which is reflected by the dichroic mirror. The peak absorption and peak emission of the sample is 495nm and 519nm respectively. Emitted light is then passed through the dichroic mirror followed by the barrier filter whose band pass wavelength is 520±18nm. Finally a small camera microscope captures the emitted wavelength, forms an image and sends to a computer.

The proposed device consists of a dichroic mirror, excitation filter, barrier filter, microscope and flashlight as its major component. These components are inexpensive and can be easily found or at parts store. Fig 1 shows the operating principle with overall block diagram of the fluorescent microscope where the white light is converted to blue light using an excitation band pass filter, and then reflected onto the sample from the surface of the dichroic mirror. The dichroic mirror has a reflection band of 380 nm to 500 nm and transmission band of 507 nm to 700 nm with reasonable intensity [11] and accuracy of more than 90% in both reflection and transmission bands. The angle of refraction of dichroic mirror is 45°. Due to this property, the dichroic mirror is also known as a beam splitter. Lastly, the barrier filter is used to allow only the desirable wavelengths. This assembly is encased in a small black box to minimize the effects of interference from the surface of the microscope. The microscope comes with an optical zoom of 20x to 200x. This helps us to zoom in to the object and take very accurate measurements. The microscope system is connected to a computer or laptop. The captured images can be saved as bmp or jpeg file format with different resolutions using the software.

**Fig 2:**
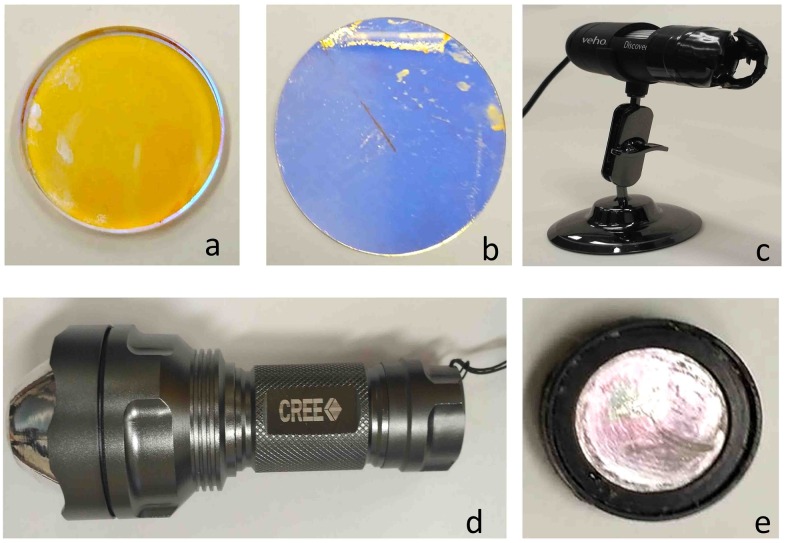
Components of the proposed microscope: (a) Dichroic mirror (b) Exciter filter (c) Pocket microscope (d) Flashlight (e) Barrier Filter.

Fig 2 shows the essential components that were used during the assembly of the proposed device. The inexpensive florescence microscope can potentially distinguish malignant cells over normal cells if properly labelled with either any tag or fluorescein.

In this work, fluorophore is used as a stimulant to differentiate between cancer and normal cells. Samples with both malignant and healthy cells were prepared by using amphotropic HEK293-derived from phoenix packaging cells to package pBabe-puro retroviral system in order to generate stable cells. The packaging cells were cultured on 100 mm cell culture plates in 10 ml of Dulbecco’s Modified Eagle’s Medium (DMEM) which was supplemented with the absorption percentage of bovine calf serum to produce retrovirus. For transfection, 10 ug of retroviral DNA was gently mixed. 430 ul of 0.15M NaCl and 60 ul of 1% Polyethylenimine (PEI) were added to the mixture followed by a gentle vortex. The solution was led to stand by for 10 minute at room temperature and was then added to the 10 mm culture plates. After 24 hour and 48 hour respectively, virus-containing supernatant was collected and filtered through 0.45 um syringe filter and was then supplemented with polybrene, and overlaid on the target cells MDA-MB-231 (a triple negative breast cancer cell lines). After 24 hour, the viral supernatant was replaced by fresh culture medium and stable expressing GFP-BRK-YF fusions were used as negative controls.

Different concentrations of fluorescein samples were made and stored in a dark place to avoid bleaching. Images from each sample were captured through the microscope on to a computer via a USB. The absorption or excitation spectrum peak and emission peak of the used fluorophore are 495 nm and 519 nm. So, we can exploit the common spectral wavelength for absorption and emission measurements. This means we need a blue light for excitation of the fluorophore and we must measure green light at the image sensor.

## Results and Discussion

**Fig 3:**
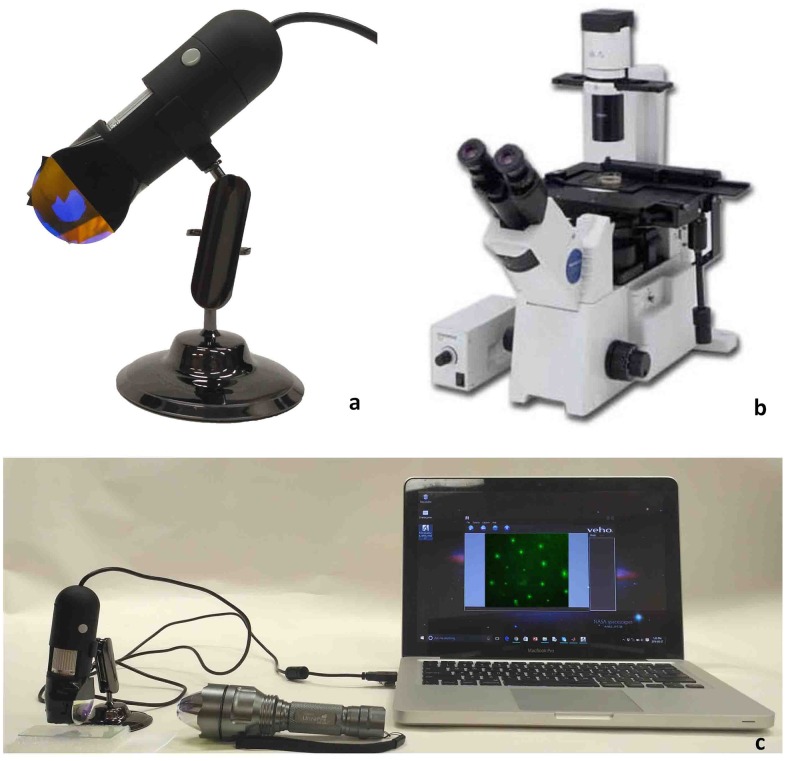
Fluorescence Microscope (a) Our prototype (b) Commercial microscope [12] (c) Experimental setup showing our proposed microscope connected to computer/laptop.

The resulting device is a small, light-weight, and portable microscope which provides an alternative to conventional fluorescence microscope with no requirement of electric power to operate (as shown in Fig 3a and 3c). This device consists of a focus knob for lens, a USB cable which is connected to the computer for viewing the image onto the computer. It is a compact and user-friendly device, as the user only needs to turn on the flashlight, run the software on computer or laptop, position the sample, and finally adjust the focus. Table 1 shows the list of components that are required to assemble the prototype along with its cost. This device has been simplified from a regular fluorescence microscope as it does not have any light path settings requirement, no external arc lamp, and no need of a filter cube selector. The captured images will be available to be viewed directly on the computer through software which is designed to be very simple and requires very low memory so that it runs smoothly on an inexpensive computer.

**Table 1:** Cost breakdown of our prototype.

Components Used	Model/Specifications	Estimated Cost ($USD)
Dichroic Mirror	DMLP490	$165.00
Excitation Filter	380–500 nm	$75.00
Barrier Filter	520±18 nm	$75.00
Microscope	Veho VMS-001 with 20x-200x magnification	$36.42
Flashlight	UltraFire C8	$6.58
Total cost of our proposed device		$358.00

Moreover, the proposed device enables us to take color images directly on computer while the commercial microscope [12] is limited to gray scale images only. The performance and features of our proposed device have been compared with a commercial device and other works [13–15]. The results are summarized in Table 2 that show superiority of our device in relation with size, weight, cost, power requirement and USB connectivity. The cost of the initial prototype of fluorescent microscope proposed in [13] was $480 [14]. Moreover, the previous work [13–15] is a mechanical system which is difficult to assemble in remote places while our prototype is easy to assemble as all the parts are available online easily at reasonable costs. Hence, anyone can build this device by buying and assembling off-the-shelf components. The proposed device also helps us in reducing the cost by 60 times in comparison to the commercial microscope (as shown in Table 2).

**Table 2:** Comparison with other devices.

	Proposed	Miller et al. [13, 14]	Babbit et al. [15]	Commercial device [12]
**Size (cm)**	11x6.5x15	7.5x13x18	Not known	56.5x29.0x57.8
**Weight (Kg)**	0.13	1	Not known	20.5
**Cost (USD)**	$358	$480 [14]	*$772.93	$23,300
**Power source**	Battery Powered	Battery Powered	Battery Powered	AC powered
**Realtime computer interface**	Yes	No	No	Yes
**Imaging mode**	Digital colour	Analog	Analog	Digital gray

* Not reported in detail; so we calculated total cost based on the list of equipment provided in the paper [15]

**Fig 4:**
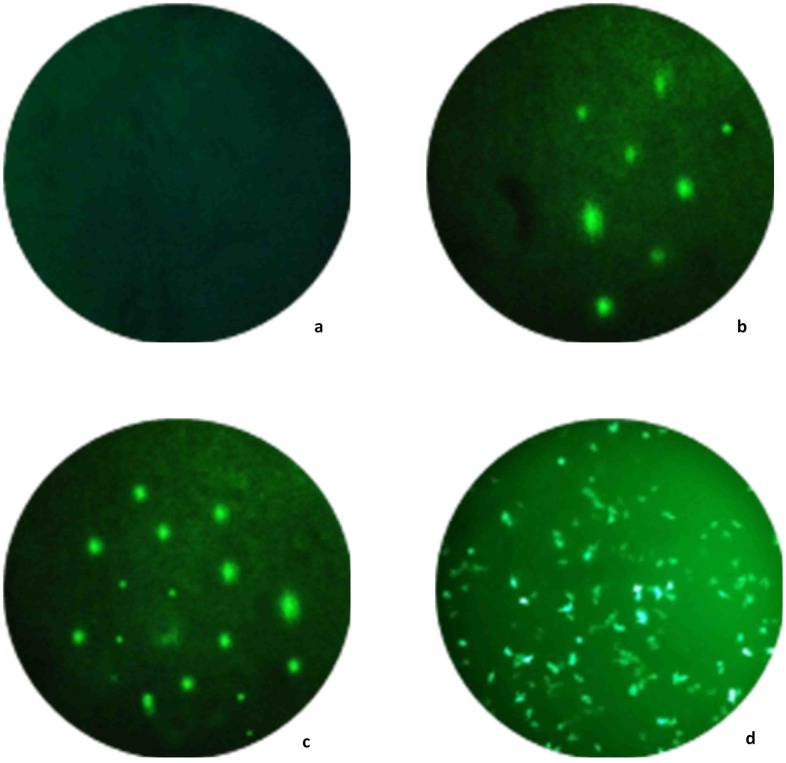
Results showing (a) Normal cell; (b)-(c) Cancer cells as viewed from our prototype; (d) Same cancer cell when viewed from the commercial microscope (model Olympus IX51).

The efficient use of the device requires some practice as focusing into the sample and placing the flashlight at appropriate position can be tricky during the initial phase. In addition to that, the software consists of a user-friendly interface which helps in configuring image parameters. The system also provides the user with an option to select the output image format, frame rate, management of the collected images, ability to focus and select a section of an image for further enhancement. The user selected configurations are saved for future use. The effect on performance of different configuration of LEDs, filters and lenses were evaluated and overall performance was also compared with the conventional fluorescent microscope. Fig 4 shows the captured image from the designed microscope as well as the commercial microscope for detecting malignant tumors (or cancer cells). It is able to differentiate between normal and cancer cells in a typical preclinical lab environment that can be easily distinguished with naked eye. The glowing parts in Fig 4b, 4c and 4d represent the cancer cells. This device is not FDA approved, and hence not intended for commercial/medical application, but designed for low-cost educational purpose only.

The application of this portable microscope is not only limited to detect cancer cells but can also be used in various other purposes as mentioned before, such as detecting water and food quality, molecular imaging and optical biopsy in gastrointestinal endoscopy.

## Conclusion

Fluorescence microscope designed in this research is a simple, yet essential, imaging technology. Commercial fluorescence microscopes are bulky, costly and requires sophisticated laboratory and trained manpower which restricts their accessibility in the regions where there are limited resources. With the aim of overcoming these limitations, we have successfully designed and tested a digital fluorescence microscope. Our design is capable of imaging micrometer-scale resolution in real-time along with controlled mechanical actuation of both the lens and sample. Combination of mechanical actuation and software into a compact and inexpensive microscope system is an important initial step towards the objective of making fluorescence microscopy universally accessible, portable, and easy to use.
